# Case report: Clinical characteristics and treatment of secondary osteoporosis induced by X-linked congenital adrenal dysplasia

**DOI:** 10.3389/fendo.2022.961322

**Published:** 2022-12-08

**Authors:** Xiaohui Tao, Tian Xu, Li Liu, Xiaoyun Lin, Zhenlin Zhang, Hua Yue

**Affiliations:** Shanghai Clinical Research Center of Bone Diseases, Department of Osteoporosis and Bone Diseases, Shanghai Sixth People’s Hospital Affiliated to Shanghai Jiao Tong University School of Medicine, Shanghai, China

**Keywords:** secondary osteoporosis, X-linked congenital adrenocortical hypoplasia, gene mutation, NR0B1 gene, efficacy of treatment

## Abstract

**Objective:**

To summarize the clinical features and bone complications in a patient from a large family with X-linked congenital adrenocortical hypoplasia (AHC) and evaluate the efficacy of different treatment regimens on the prognosis of secondary osteoporosis caused by AHC at a 5-year follow-up.

**Methods:**

A large family with AHC was recruited, and the causative gene mutation was identified by Sanger sequencing in the proband. Clinical features as well as radiological examinations and laboratory indices of osteoporosis secondary to AHC were analyzed in this study. Meanwhile, the proband was treated with classical antiresorptive drugs (bisphosphonates) for 2 years and switched to a vitamin K_2_ analogue for another 3 years, during which the efficacy of the drugs was evaluated.

**Results:**

The proband was identified as carrying a homozygous insertion mutation (p. Thr193GlyfsX13) in the *NR0B1* (nuclear receptor subfamily 0, group B, member 1) gene, resulting in a premature stop codon due to a frameshift mutation. During treatment and follow-up, the proband did not respond well to bisphosphonate and developed atypical femoral fractures. Vitamin K_2_ improved clinical symptoms. In terms of bone mineral density (BMD), there is no evidence of any effect of vitamin K_2_ on the neck of femur, though some minor effects on spinal BMD cannot be excluded.

**Conclusions:**

Secondary osteoporosis induced by AHC deserves clinical attention. Unlike in primary osteoporosis, the curative effect of bisphosphonates was unsatisfactory and was more likely to cause atypical femoral fractures in long-term treatment. It is suggested that bone anabolic drugs may be better alternatives.

## Introduction

Osteoporosis is characterized by low-energy fractures resulting from low bone mass and damaged bone microarchitecture. It is especially prevalent in postmenopausal women and older men, and can be evaluated by dual-energy x-ray absorptiometry (DXA). It is crucial to distinguish primary from secondary osteoporosis, which directly affects the choice of treatment options, appraisal of therapeutic efficacy, the incidence of side effects, and prediction of prognosis. Secondary osteoporosis occurs in almost 2/3 of men, more than half of premenopausal and perimenopausal women, and approximately 1/5 of postmenopausal women (1). Multiple diseases or drugs affecting bone metabolism can lead to secondary osteoporosis, of which hypogonadism is the most common cause in men (2, 3).

In this study, we report an adult patient with osteoporosis secondary to congenital adrenal dysplasia complaining of low BMD. Congenital adrenal dysplasia is a rare disorder inherited in X-linked (OMIM 300200) and autosomal recessive (OMIM 240200) forms that also occurs as part of the Xp21 deletion syndrome. Mutations in the *NR0B1* gene mapped on chromosome Xp21 are responsible for AHC (4). *NR0B1* encodes the DAX-1 (dosage sensitive sex reversal, adrenal hypoplasia, critical region on the X chromosome, gene 1) protein, which is mainly expressed in steroidogenic tissue (gonads and adrenal cortex), as well as the hypothalamus and pituitary. It plays an essential role in the development and regulation of the adrenal gland and reproductive axis (5).

AHC has an approximate incidence of 1:12,500 live births in Australia (6). A study from the UK demonstrated that the incidence rate in children is 1:140,000–1:1,200,000, of which the incidence rate in male patients was 1:70,000–1:600,000 (7). Furthermore, AHC displayed a two-peak profile divided by the age of onset (4). The majority of patients with AHC have an early onset, usually within 2 months of life, accompanied by severe salt wasting and high mortality. Late onset occurs in the age range of 2 to 9 years, and clinical symptoms are more atypical and insidious (4, 8, 9). In general, male patients with X-linked AHC usually present primary adrenal insufficiency (PAI) or isolated mineralocorticoid deficiency at an early age, with or without hypogonadotropic hypogonadism (HHG) (10), while female patients are almost always asymptomatic carriers.

Apart from typical clinical symptoms, such as PAI and HHG, attention should be given to skeletal complications such as osteoporosis, which is easily overlooked. The mechanism of osteoporosis secondary to AHC is still not fully elucidated, and hypogonadism is one of the contributors (11). In the present study, the AHC patient with severe osteoporosis had been followed up for almost 5 years. A poor response to bisphosphonates was observed, and atypical femoral fractures (AFFs) occurred during the first 2 years of treatment. Given the primary disease and concomitant therapy of steroids in this patient, it was important to choose an optimal therapeutic regimen.

Here, the clinical characteristics of osteoporosis secondary to AHC are analyzed, and the choice of drugs for such diseases is discussed. The purpose of this case report is to attract the attention of clinicians to select appropriate drugs, avoid serious complications, and obtain the best curative effect.

## Materials and methods

### Subject

**Figure 1:**
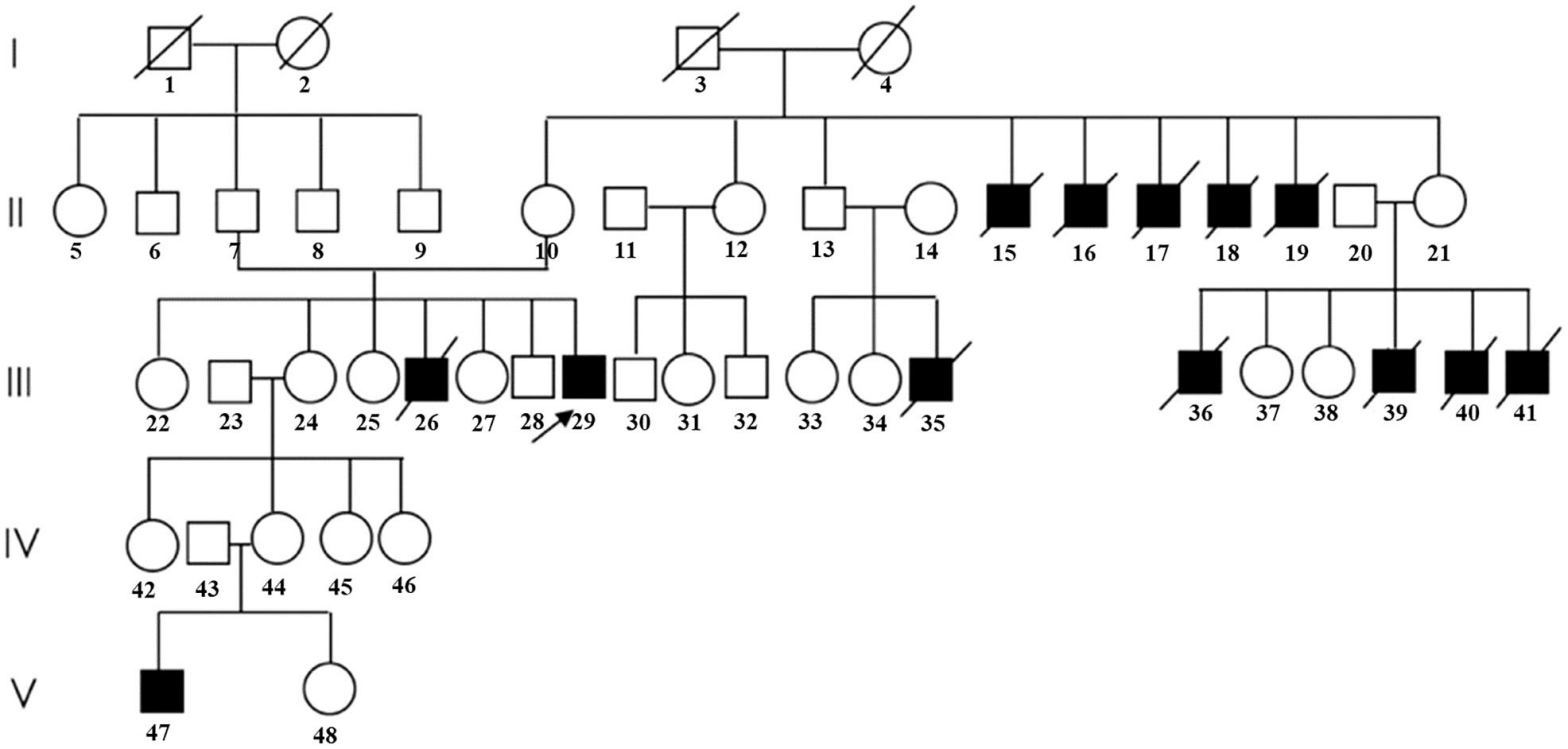
Pedigree of the family in the present study. (Patients with typical clinical symptoms of AHC are shown by darkened symbols. The black arrow indicates the proband.).

The present study was approved by the Ethics Committee of Shanghai Sixth People’s Hospital Affiliated with Shanghai Jiao Tong University School of Medicine. We investigated a middle-aged male patient from an AHC family who complained of low BMD (**Figure 1**). Written informed consent was obtained from the participant.

### Clinical, laboratory, and imaging evaluation

Anthropology measurements (including height, weight, and age) and detailed medical history were recorded. Complete medical check-ups were performed, including skin pigmentation and secondary sexual signs. Blood samples were extracted for routine biochemical indicators, sex hormones, and bone turnover markers (BTMs). Radiographs of the thoracolumbar spine, abdominal computed tomography (CT), cerebral magnetic resonance imaging (MRI), and bone scintigraphy were performed. BMD (g/cm^2^) of the lumbar spine 1–4 (L1–4), left femoral neck, and total hip was measured by Lunar Prodigy DXA (GE Healthcare, Madison, USA). The LSC (least significant change) was calculated according to International Society for Clinical Densitometry (ISCD) recommendations (12). To be considered significant, the BMD difference before and after medication should be greater than the LSC. The LSC (lumber spine, femur neck, and total hip) at our center was 0.028 g/cm^2^.

### Genetic analysis

Genomic DNA was extracted from peripheral blood using a conventional method. The primers were designed by Primer3 software (http://bioinfo.ut.ee/primer3-0.4.0/, **Supplementary Table 1**). After predenaturation at 95°C for 2 min, PCR amplifications were performed for 35 cycles (96°C for 10 s, 68°C for 1 min, and 72°C for 1 min). PCR products were purified and sequenced on an ABI3730XL platform with the BigDye3.1 Kit (ABI company, USA).

## Case report

Proband III-29 (**Figure 1**), a 46-year-old man, was born at term from an uneventful pregnancy with a birth weight of 3.6 kg, and the parents were nonconsanguineous, healthy, and of average height. By age 8, he developed recurrent episodes of fever, chills, and fatigue with unknown causes. Growth retardation was observed, and hyperpigmented macules grew, especially on the lips, ears, and joints of the fingers. Immediately, he was diagnosed with PAI (specific data were not available) at the local hospital and treated with prednisone for nearly 30 years, with a maximum dose of up to 30 mg per day maintained until the present. During this time, acute adrenal crises occurred twice due to improper drug withdrawal. Due to the lack of secondary sexual characteristics, HHG was diagnosed, and testosterone undecanoate was administered 10 years ago, during which he recovered his sexual function slightly. Backache, fatigue, and muscle cramps gradually developed at the age of 35. Alendronate was administered for 4 years and 10 months, withdrawn for 3 years, and then restarted for 2 years. However, the treatment effect was not satisfactory, and neither the bone pain nor the BMD improved, so he came to our clinic for help (specific data were not available).

**Figure 2:**
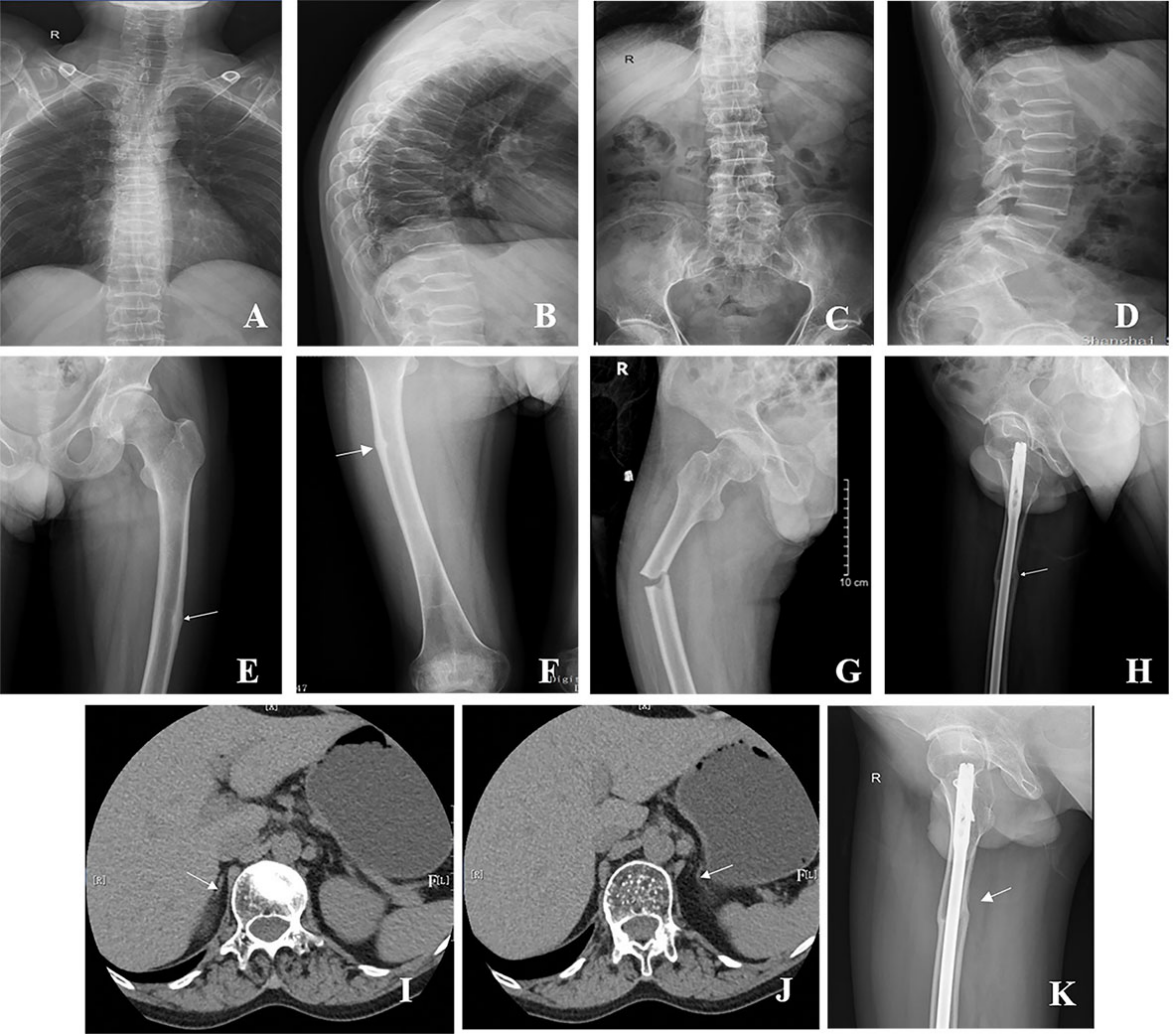
Radiographs of the patient with AHC. **(A–D)** Multiple vertebral compression fractures. **(E, F)** The white arrow shows the pseudofracture line in the left and right femurs. **(G)** Right femoral shaft fracture at the site of the pseudofracture line. **(H)** Right femoral shaft fracture after internal fixation. **(I, J)** The white arrow shows that the adrenal glands on both sides were small, and the structure was unclear. **(K)** Right femoral shaft fracture healing in 2022.

At his first visit to our clinic, he mentioned that his family members (III-26, 35, 36, 39, 40, and 41) also presented the same symptoms as him, including skin pigmentation and salt wasting. Unfortunately, all of them passed away at ages ranging from 7 to 11. Another patient (V-47) was 14 years old and presented with weight loss and progressive skin pigmentation. The height, fingertip distance, and weight of the proband were 156 cm (−4.5 SD), 168 cm, and 48 kg, respectively. Physical examination revealed moderate hyperpigmentation on the buccal mucosa, lips, and gums; normal olfaction; sparse pubic hair; small penis; and low testicular volumes of 4 ml on the left and 5 ml on the right (Tanner stage 2). Imaging examination indicated multiple compression fractures of the thoracolumbar spine and crumpled adrenal glands without calcification (**Figure 2**), which were detected by x-ray, bone scintigraphy, and abdominal CT scans. To evaluate the changes in the hypothalamus and pituitary, the patient was examined by brain MRI without any abnormalities. In addition, DXA revealed a low BMD (L1–L4, *Z* score −2.3 SD; femoral neck, *Z* score −2.9 SD; total hip, *Z* score −2.6 SD).

Laboratory parameters are summarized in **Supplementary Table 2**. As a clinical sign of PAI, the patient showed low levels of serum cortisol at 8 o’clock in the morning and elevated serum levels of ACTH. The plasma concentrations of renin supine and angiotensin II exceeded the upper limit of the normal reference value, while aldosterone was below the lower limit of the normal reference value. The patient exhibited low serum levels of T and LH. Sanger sequencing of *NR0B1* revealed an inserted mutation (c.572_575dup) in exon 1, resulting in a frameshift with premature termination (p.Thr193GlyfsX13, **Supplementary Figure 1**). Combined with a molecular diagnosis, clinical characteristics, and laboratory indices, the diagnosis of AHC was established.

**Figure 3:**
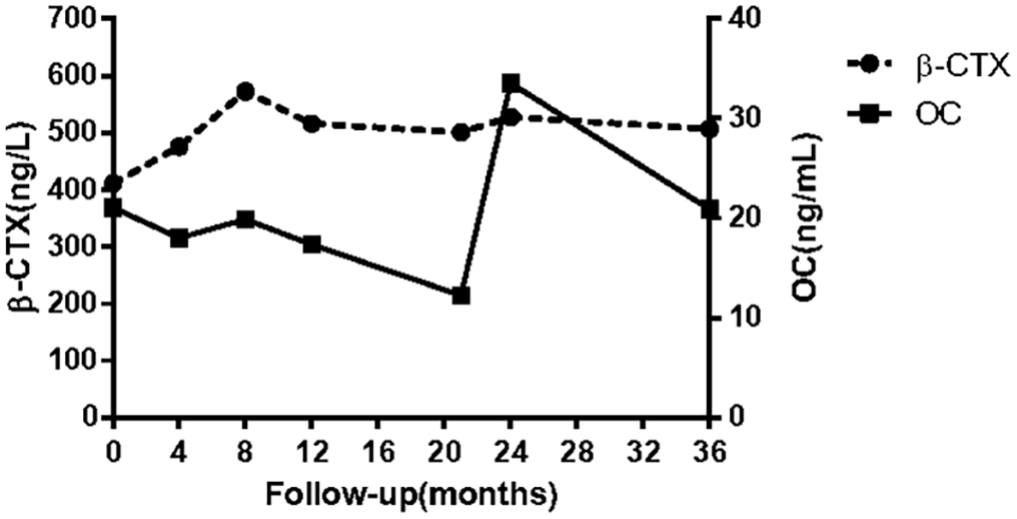
Bone turnover markers during treatment (alendronate orally at the beginning of treatment; a single infusion of intravenous zoledronic acid by the 12th month; right femur fracture by the 14th month; atypical fracture of left femur by the 20th month; menatetrenone orally by the 21st month). β-CTX: β-isomerized C-terminal telopeptide of type I collagen; OC: serum osteocalcin in the form of an N-terminal mid-molecule fragment.

Given the high levels of BTMs and poor efficacy probably resulting from the intermittent use of bisphosphonates, a regular oral alendronate treatment (70 mg, alendronate sodium, and vitamin D3 tablets, Merck Sharp & Dohme Ltd, USA) was advised. The BTMs and BMD (**Table 1**) were rechecked 1 year later, and the results indicated that the effect of oral drugs was not ideal even when taking them regularly. Therefore, intravenous zoledronate (5 mg, Aclasta, Novartis Pharma Stein AG, Switzerland) yearly was recommended in the next year. After treatment with zoledronic acid for nearly 1 year, the patient complained of pain in the right proximal femur, and a pseudofracture line was detected by x-ray (**Figure 2E**). One month later, an AFF occurred in the right femur at the site of the pseudofracture line due to an accidental fall, and internal fixation was performed (**Figure 2H**). A pseudofracture line in the middle of the left femur and a significant decrease in BMD of the femur neck were also found in the same year. Based on the conditions described above, bisphosphonates were not appropriate for the patient, and treatment was changed to vitamin K_2_ analogues (Menatetrenone, Eisai, Japan, 15 mg tid p.o.). During the subsequent follow-up, the level of serum osteocalcin was significantly elevated, bone pain was relieved, and no pathological fractures appeared (**Figure 3**). Subsequently, BMD in the lumbar spine 1–4 and total hip increased from 2019 to 2022 (**Table 1**). The difference in BMD in the lumbar spine and total hip from 2019 to 2022 exceeded the LSC (0.077 and 0.082 g/cm^2^ compared with 0.028 g/cm^2^). However, the difference in BMD in the neck of the femur from 2019 to 2022 was below the LSC (0.007 g/cm^2^ compared with 0.028 g/cm^2^).

**Table 1:** The change in bone mineral density.

Date	Lumbar 1–4		Femur neck		Total hip	
	g/cm^2^	*Z*-score (SD)	g/cm^2^	*Z*-score (SD)	g/cm^2^	*Z*-score (SD)
2017-8	0.787	−2.3	0.526	−2.9	0.578	−2.6
2018-8	0.792	−2.3	0.604	−2.2	0.579	−2.6
2019-10	0.824	−2.0	0.551	−2.6	0.563	−2.7
2020-9	0.879	−1.7	0.623	−2.2	0.597	−2.6
2022-10	0.901	−1.1	0.558	−2.5	0.645	−2.5

2017-8: at first visit and continuing to take alendronate regularly; 2018-8: switching to intravenous zoledronate; 2019-10: starting to take menatetrenone.

## Discussion

AHC, a special type of PAI, was first documented by the pathologist Sikl in 1948 (8). In 1994, Muscatelli et al. found that the candidate gene was localized at the Xp21.3 region (13). The *NR0B1* gene consists of two exons separated by a single intron and encodes the DAX-1 protein with a length of 470 amino acids, an orphan member of the nuclear receptor superfamily (14). Instead of the highly conserved zinc finger DNA binding domain (DBD) of other classical nuclear receptors, the N-terminal of the DAX-1 protein contains 3.5 alanine/glycine-rich repeats of a 65–70 amino acid motif, which plays a vital role in the transcriptional regulation of *NR0B1* (15–17). In contrast, the highly conserved C-terminal domain is homologous to the ligand-binding domain (LBD) of the nuclear receptor superfamily and mediates ligand binding, dimerization, and nuclear localization (10).

To date, over 300 individuals and families with AHC have been reported, most of which harbor frameshift or nonsense mutations (18–22). It was speculated that the hot spot mutation of *NR0B1* was located in the putative LBD, causing premature truncation of the DAX-1 protein (7, 22). Previous studies indicated that clinical phenotypic heterogeneity was detected in patients with AHC, even when caused by the same mutation of the *NR0B1* gene (23, 24). Affected individuals in this pedigree manifested various clinical phenotypes, which supported the above conclusion and hinted at the important role of genetic heterogeneity and epigenetic factors in the phenotype and prognosis of AHC. Further studies are needed to reveal the complex genotype–phenotype correlation.

Bertalan reported two brothers with serious osteoporosis secondary to AHC at the ages of 51 and 43 and revealed two novel *NR0B1* mutations (c.568-571del and c.572-575del). However, the treatment method was not mentioned (25). Previous studies did not focus on osteoporosis secondary to AHC. In fact, it is an inevitable complication of AHC with serious consequences. Our study focused on osteoporosis secondary to AHC for the first time and discussed the choice of effective treatment.

This case is not simple secondary osteoporosis and has many clinical and biochemical characteristics different from classical GIOP. The long-term effect of GC on bone metabolism is the inhibition of osteogenesis. Theoretically, the BTMs of the patient should decrease. However, the bone metabolism of this patient was not inhibited, and treatment with bisphosphonates failed to effectively reduce the level of BTMs. At the same time, we did not expect that AFFs would occur when the BTMs were not suppressed. The mechanism of osteoporosis secondary to AHC has not been fully elucidated. One explanation was that severe osteoporosis was induced by the lack of a multitude of hormones, especially androgen (26). However, some studies reported patients with HH accompanied by osteoporosis or osteopenia, where bone loss was irreversible even with sufficient androgen substitution (27–29). On the other hand, long-term corticosteroid replacement therapy of this patient also had an adverse impact on BMD (30). Therefore, choosing an appropriate drug is the key point for the prevention and treatment of osteoporosis secondary to AHC.

Antiresorptive drugs, such as alendronate or zoledronate, are classical anti-osteoporosis medications that show satisfactory therapeutic effects in most patients with osteoporosis (31). To evaluate the efficacy of drugs for osteoporosis, BTMs are often measured in clinical practice (32). In this patient, long-term high levels of BTMs (OC and β-CTX) indicated that the efficacy of bisphosphonates, either oral or intravenous, was somewhat disappointing. More seriously, AFFs occurred during the treatment. Generally, bisphosphonates are effective in reducing the risk of osteoporotic fractures, and the risk of AFF increases gradually with the length of treatment. However, it usually occurs after continuous use for more than 5 years (33). Moreover, a cohort study indicated that women with osteoporosis who took bisphosphonate holidays were less likely to sustain AFFs (0.03%) than women in the persistent and nonpersistent groups (0.15%). The average drug holiday was 3.1 ± 1.6 years, which occurred after 5.2 ± 1.8 years of bisphosphonate use, and medication was restarted for an additional mean time of 2.5 ± 2.0 years (34). The patient in our study received 4 years of continuous treatment after a 3-year bisphosphonate holiday; not only was the drug effect unsatisfactory, but serious adverse events also occurred. There might be several possibilities to explain the case. First, despite a 3-year drug holiday, the cumulative duration of medication was too long to attenuate its efficacy. Second, the *NR0B1* gene with the mutation site (c.572_575dup) encodes a truncated protein, which is related to bone metabolism and the function of osteoclasts and osteoblasts through an unknown mechanism. Hence, further pharmacogenomic analysis and functional research both *in vitro* and *in vivo* need to be performed.

Long-term hormone replacement and symptomatic treatment were often administered in AHC patients. Before puberty, male patients with HHG were treated with androgen to develop secondary sexual characteristics and improve their quality of life. Previous studies indicated that bone loss was irreversible even after sufficient testosterone replacement after puberty (28, 35). Therefore, we hypothesized that the bone health of AHC patients should be considered before skeletal maturation. Furthermore, with a diagnosis of osteopenia or osteoporosis, it is necessary to closely monitor the patient and be cautious about side effects, such as AFF and osteonecrosis of the jaw (ONJ), when using anti-osteoporotic drugs, especially bisphosphonates (33).

Anabolic drugs are another type of medication that act through changes in bone remodeling, bone modeling, or both and an increase in bone formation (36). Teriparatide, as a classical anabolic drug, is not ideal in patients at high risk of femoral neck or other cortical fractures. In addition, teriparatide is not approved for male patients or secondary osteoporosis in China. Given the above, the patient was advised to take menatetrenone instead. Menatetrenone has been proven to reduce osteoporotic fractures and improve hip bone strength despite its modest effect on BMD (37, 38). The mechanism of menatetrenone aims to promote osteocalcin γ-carboxylation, and only carboxylated osteocalcin has a weak osteogenic effect, which explains its mild effect on bone formation and increases in the osteocalcin level (37). After 3 months of treatment, the patient presented with greatly relieved bone pain, and the level of osteocalcin (bone formation marker) had increased substantially.

There is an explanation for the inconsistent changes in BMD in different bone sites after treatment. When this patient was initially treated with alendronate, the BMD of the femoral neck increased significantly (*Z* score from −2.9 to −2.2), which indicated that bisphosphonate treatment was effective. Because zoledronic acid is more potent than alendronate, we administered zoledronic acid to increase BMD and reduce BTMs. However, unexpectedly, atypical fractures occurred. Improvement in the BMD of the femoral neck requires weight-bearing and mechanical stimulation in addition to drug effects. Due to the AFF, lower extremity pain led to limited movement of the patient, which was also the reason for the decrease in BMD in the femoral neck (*Z* score from −2.2 to −2.6). After surgical treatment, his mobility was significantly improved and combined with a bone formation agent, he had an increased BMD in his femoral neck (*Z* score from −2.6 to −2.2). However, *Z* score dropped to −2.5 by 2022, and therefore there is no evidence of any effect of vitamin K on the neck of the femur.

In conclusion, the present study described the clinical features and bone complications in a male patient with secondary osteoporosis induced by AHC who was followed up for 5 years and adjusted anti-osteoporosis treatments three times. It is suggested that for adult patients with AHC, close attention should be paid to skeletal complications, especially when combined with glucocorticoid therapy. It is of great importance to evaluate bone mass and provide treatment as early as possible, accompanied by regular follow-up. Furthermore, anabolic agents may be a better choice for the treatment of osteoporosis secondary to AHC.

## Data availability statement

The datasets presented in this study can be found in online repositories. The names of the repository/repositories and accession number(s) can be found in the article/**Supplementary Material**.

## Ethics statement

The studies involving human participants were reviewed and approved by the Ethics Committee of Shanghai Jiao Tong University school of medicine Affiliated Sixth People’s Hospital. The patients/participants provided their written informed consent to participate in this study.

## Author contributions

XT and TX drafted the manuscript. LL, XL, XT, and TX collected follow-up data. ZZ, HY, XT, and TX revised the manuscript. All authors contributed to the article and approved the submitted version.
