# Supplementary material for: Case report: Clinical characteristics and treatment of secondary osteoporosis induced by X-linked congenital adrenal dysplasia
Source: Front Endocrinol (Lausanne). 2022 Dec 8;13:961322. doi: 10.3389/fendo.2022.961322 (PMC9772268; doi:10.3389/fendo.2022.961322)
Supplement: Supplementary file 1 [file DataSheet_1.pdf]

Supplementary table 1. The primers sequence of the *NR0B1* gene

Fragments	Primers (5′–3′) sense
NR0B1-1F	TAGAGCAGTGCTTTTCAAACGTC
NR0B1-1R	GGTGCTCTTTAAAAGCTGGAAATG
NR0B1-2F	GATGCGAGGGTTCAATGGAAAGAGTT
NR0B1-2R	CACGTGCGTTTGCTTTGAGCTAGTG
NR0B1-3F	AAACGTACGCGGCACCGAAG
NR0B1-3R	AGCAGTTGCGCACCAGCAC
NR0B1-4F	GCGTGCCCACGAGCACAA
NR0B1-4F	GCGTGCCCACGAGCACAA
NR0B1-4R	ACTGCCCGCGCCCCTAGATA
NR0B1-5F	ACTGGTTTGGCCTTTTACCCTTT
NR0B1-5R	GCTACCTGTTGGCAAATGTCTTC

Supplementary table 2 | Laboratory findings in the proband with AHC

Indicators	Baseline value	Normal reference
Na^+ (mmol/L)	139	135~145
K^+ (mmol/L)	3.9	3.5~5.5
Ca^{2+} (mmol/L)	2.35	2.08~2.60
P^- (mmol/L)	1.31	0.80~1.60
Cortisol at 08:00 h (μg/dL)	1.19	6.20~19.4
ACTH (ng/L)	117.20	7.2~63.3
PRC supine at 08:00 (μIU/mL)	>500.0	2.8~39.9
AngII (pg/mL)	189	25-60
ALD supine at 08:00 (ng/dL)	2.29	1.17~23.6
T (nmol/L)	3.82	8.40~28.70
LH (IU/L)	0.26	1.4~18.1 (male 13-70 y)
FSH (IU/L)	5.79	1.5~9.3 (male 20-70 y)
OGTT 2h (nmol/L)	3.66	3.90-7.80
β-CTX (ng/l)	411.10	< 584 (male 30-50 y)
OC (ng/ml)	21.06	14~42 (male 30-50 y)
25OHD (ng/mL)	49.47	>20
ALP (U/L)	114	15-112
PTH (pg/ml)	27.50	15.00~65.00

ABBERVATIONS: ACTH: adrenocorticotropic hormone; PRC: plasma renin concentration; AngII: angiotensin II; ALD: aldosterone; T: testosterone; LH: luteinizing hormone; FSH: follicle-stimulating hormone; OGTT 2h: 2 hours after a 75-g oral glucose tolerance test; β-CTX: β-isomerized C-terminal telopeptide of type I collagen; OC: serum osteocalcin in the form of an N-terminal mid-molecule fragment; ALP: alkaline phosphatase; PTH: parathyroid hormone

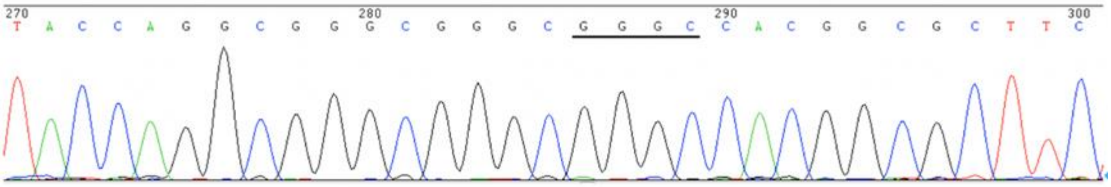

Supplementary Figure 1 | Sequencing traces of the mutation in the *NR0B1* gene (a homozygous c.572_575dup insertion in exon 1 of *NR0B1*, resulting in a frameshift p.Thr193GlyfsX13)
